# EccDNA Analysis Provides Novel Insights Into the Molecular Mechanism of Firmness of Fish Fillet

**DOI:** 10.1002/fsn3.70268

**Published:** 2025-05-13

**Authors:** Kai Zhang, Jianchao Chen, Haobin He, Binwei Duan, Canbei You, Zehua Hu, Linhao Cai, Xi Xiang, Rishen Liang

**Affiliations:** ^1^ College of Life Sciences and Oceanography Shenzhen University Shenzhen China; ^2^ College of Animal Science and Technology Zhongkai University of Agriculture and Engineering Guangzhou China; ^3^ Scientific Research Center The Seventh Affiliated Hospital of Sun Yat‐Sen University Shenzhen Guangdong China

**Keywords:** extrachromosomal circular DNA, fillet quality improvement, muscle texture

## Abstract

Extrachromosomal circular DNAs (eccDNAs) play a significant role in regulating various biological processes, including abnormal muscle development. The molecular functions and impact of eccDNAs in the muscle development of fish are poorly understood. To investigate the potential roles of eccDNAs in the muscle development of fish, we analyzed and compared the expression profile of muscle eccDNAs of crisp grass carp, fed a faba bean meal‐based diet, and ordinary grass carp, fed a practical diet. Using the Circle‐seq strategy, we found the eccDNA abundance in crisp grass carp (211,920 eccDNAs) was significantly higher than that in ordinary grass carp (25,857 eccDNAs), suggesting that the faba bean diet likely independently influences eccDNA production. Compared to ordinary grass carp, crisp grass carp exhibited 10,565 upregulated and 129 downregulated eccDNAs, indicating eccDNAs were possibly associated with the muscle development of grass carp. GO and KEGG enrichment analyses indicated that the upregulated eccDNAs were related to muscle fiber development, cellular structure, and cell junctions. Based on our results, we speculated that the overexpression of genes involved in muscle fiber, calcium metabolism, and collagen driven by eccDNAs likely contributes to the observed increase in muscle fiber density, calcium levels, and collagen content in crisp grass carp, thereby enhancing muscle hardness. Notably, eccDNAs were identified as potential innate immunostimulants capable of eliciting immune responses in fish. In summary, our findings demonstrate that eccDNAs are aberrantly expressed in the muscles of fish fed a faba bean diet, offering novel insights into the molecular mechanisms underlying muscle hardening in fish.

## Graphical abstract

We provide a new and basic eccDNA database for the molecular regulation of muscle firmness in grass carp. The eccDNA abundance in crisp grass carp was significantly higher than that in ordinary grass carp. EccDNAs likely play important roles in the muscle‐hardening of crisp grass carp.

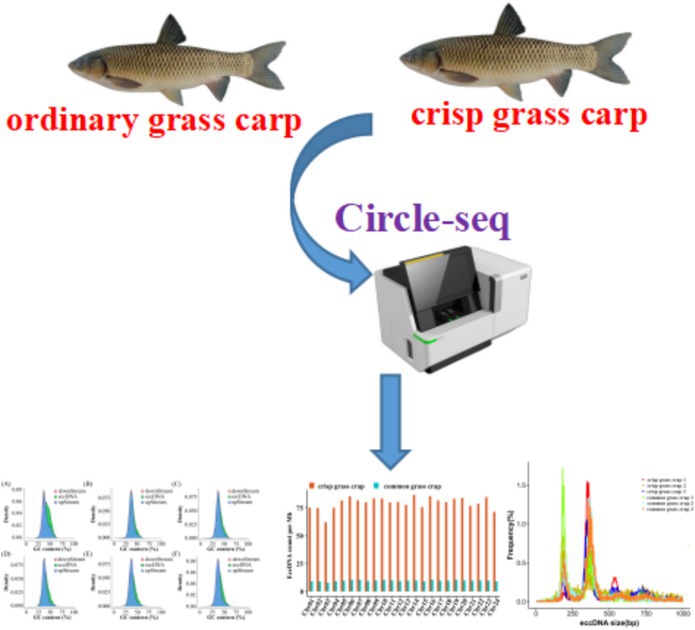

## 1 Introduction

Muscle texture is a critical determinant of fish quality, directly influencing the production of high‐quality fish products (Hyldig and Nielsen 2001). Understanding the molecular mechanisms regulating fish muscle texture could significantly advance the development of premium fish products and enhance profitability in the aquaculture industry (Elvevoll et al. 1996). Among muscle characteristics, hardness, or firmness, is particularly important in shaping consumer preferences (Veland and Torrissen 1999). Grass carp (*Ctenopharyngodon idellus*), a freshwater fish indigenous to China, holds significant economic importance (Xu et al. 2020), with production exceeding 5.7 million tonnes in 2022, accounting for one‐fifth of China's freshwater aquaculture output. A notable experiment demonstrated that feeding grass carp with faba bean ( *Vicia faba* ) for 90–120 days significantly improved the crispness and firmness of their muscle texture (Yu et al. 2017; Hao et al. 2024). This led to a rise in the popularity of crisp grass carp in China, with its products successfully penetrating markets in the United States, Southeast Asia, and Latin America. Moreover, the diameter and density of muscle fibers have been highly correlated with muscle hardness (Johnston et al. 2000; Yu et al. 2017). Compared to ordinary grass carp, crisp grass carp exhibit reduced muscle fiber diameter and increased fiber density (Yu, Xie et al. 2014; Feng et al. 2016; Tang et al. 2024). Interestingly, while their immediate muscle composition (lipid, water, or protein) shows no significant differences (Tian et al. 2020; Hao et al. 2024; Ma et al. 2024), crisp grass carp display higher collagen content and smaller muscle fibers (Ma et al. 2020). Despite prior studies identifying several proteins and genes potentially linked to muscle firmness, the molecular mechanisms driving increased muscle hardness in crisp grass carp remain poorly understood (Yu et al. 2020; Fu et al. 2022; Tian et al. 2023).

Extrachromosomal circular DNAs (eccDNAs) are unique DNA molecules characterized by their double‐stranded and circular architecture. They originate from genomic DNA and exist independently of chromosomal DNA (Wang et al. 2021). Ranging in size from hundreds of bases to megabases, eccDNAs are prevalent in eukaryotes, including yeast, nematodes, fruit flies, plants, and mammals (Møller et al. 2016; Kumar et al. 2017). EccDNAs can replicate autonomously and deviate from Mendelian inheritance, with their uneven distribution in daughter cells enhancing gene transcription efficiency compared to chromosomal DNA (Wu et al. 2019; Lv et al. 2024). They possess the capacity to affect phenotypes by adjusting the quantity of gene copies and the transcription of full‐length or truncated genes (Nathanson et al. 2014; Paulsen et al. 2018; Zhu et al. 2021). Moreover, eccDNA harboring regulatory elements, such as an enhancer, can regulate gene amplification through intramolecular interactions (Sheng et al. 2024). Emerging evidence suggests that eccDNAs are associated with the regulation of muscle development. For instance, the highest quantity of eccDNAs has been found in the human muscle protein‐coding gene titin (TTN), indicating that eccDNA may aid host cells in carrying out their intended role (Møller et al. 2018). Similarly, eccDNA derived from the AGRIN gene, enriched in king pigeon muscle, encodes membrane proteins essential for neuromuscular junction development, with mutations leading to abnormal muscle development (Møller et al. 2020). Recently, we revealed that eccDNAs involved in muscle characteristics were highly abundant in the muscle cells of slimming grass carp compared to those of ordinary grass carp, suggesting that the enriched eccDNAs might have an effect on the activation of muscle firmness (He et al. 2024).

Despite these findings, the role of eccDNAs in fish muscle development remains unclear. This study aims to explore the eccDNAs present in the muscle of grass carp fed on faba beans, exploring their role in regulating fish muscle texture. The findings provide a foundation for improving fish fillet quality in aquaculture.

## 2 Material and Method

### 2.1 Samples Collecting and DNA Extraction

A total of 120 fish were randomly allocated into ordinary grass carp and crisp grass carp groups, with three replicates per treatment group. They were raised in six tanks (20 fish in each tanks) at an aquatic farm in Zhongshan, Guangdong, China. Grass carp fed a practical diet were referred to as ordinary grass carp, while those fed exclusively with faba bean ( *Vicia faba* ) meal were termed crisp grass carp. The composition of the practical diet was consistent with that used in the study by Gan et al. (2017). Both groups were reared under conventional aquaculture conditions (a water temperature of 25°C, pH = 7.0, and dissolved oxygen of 5 mg/L). The final weight was approximately 4.5 kg for the crisp grass carp group and approximately 5 kg for the ordinary grass carp group after three months. The muscle texture of ordinary grass carp is relatively soft, whereas that of crisp grass carp is significantly firmer and harder, allowing the two to be distinguished. Three ordinary grass carp and three crisp grass carp were randomly selected and euthanized by immersion in eugenol (80 mg/L). Approximately 10 g of dorsal muscle tissue was excised from each sample and promptly cryopreserved in liquid nitrogen. The extraction of genomic DNA was carried out using the MagAttract HMW DNA Kit (QIAGEN), according to the manufacturer's instructions. We assessed the quality of the extracted DNA through electrophoresis on 1% ethidium bromide‐stained agarose gels. DNA concentration and purity were measured using a NanoDrop Microvolume Spectrophotometer (Thermo Scientific).

### 2.2 Removal of Linear DNA

To enrich circular DNA, residual linear genomic DNA was removed from each sample. The DNA was digested at 37°C for 120 h using Plasmid‐Safe ATP‐dependent DNase (Biosearch, E3110K), following the manufacturer's protocol. Additional DNase and ATP were added every 24 h. The effectiveness of linear DNA removal was verified through PCR amplification of the circular DNA marker gene (*COX3*) and the linear DNA marker gene (*NDL6*) PCR amplification was performed with an initial denaturation at 94°C for 5 min, followed by 35 cycles of denaturation at 94°C for 30 s, annealing at 55°C (*COX3*) or 62°C (*NDL6*) for 30 s, and extension at 72°C for 60 s. A final extension was conducted at 72°C for 5 min. The PCR products were evaluated via agarose gel electrophoresis. The presence of a specific *COX3* band and the absence of an *NDL6* band confirmed the complete removal of linear genomic DNA.

### 2.3 Circular DNA Enrichment

Following linear DNA removal, circular DNA was amplified randomly using Phi29 polymerase and exonuclease‐resistant random primers through rolling circle amplification (RCA). The RCA reaction was carried out at 30°C for 72 h. The amplified eccDNA was purified using a Cycle‐Pure Kit (Omega) and digested with *NdeI* (Thermo Fisher Scientific) to verify RCA results before sequencing.

### 2.4 Circular DNA Identification

The Circle‐Map programme (Prada‐Luengo et al. 2019) was used to identify the eccDNAs. To increase accuracy, filtering parameters included: “(1) Circle score >=200, (2)Split‐read mapping>= 2, (3)Start site coverage enhancements >= 0.33, (4) Terminal coordinate coverage enhancements >= 0.33, (5) Sequencing coverage uniformity <= 0.1, and (6) Mean coverage >Standard deviation”. For further examination, all eccDNAs that met the filtering requirements were utilized.

### 2.5 EccDNA Source Region and Annotation

To analyze the genomic distribution of eccDNAs, each chromosome was divided into 50 kb windows, and the abundance of eccDNAs within each window was quantified. A Manhattan plot was generated to visually represent the distribution. Protein‐coding gene sequences from the grass carp reference genome were utilized (Wu et al. 2022). BEDtools was employed to identify overlaps between annotated gene sequences and eccDNA coordinates (Quinlan 2014). Custom R scripts were utilized to screen annotated eccDNA genes, retaining those with overlapping base pair numbers exceeding 60 for further analysis.

### 2.6 Differential Analysis of Circular DNA Expression

The tag count data of eccDNAs was analyzed using the edgeR software for differential expression analysis (Robinson et al. 2010). The analysis included three key steps: (1) normalization of the tag count; (2) computation of probability of hypothesis testing (*p*‐value), depending on the model; and (3) multiple hypothesis testing and correction to determine the FDR value (error detection rate). eccDNAs with a *p*‐value < 0.05 and |log_2_FC| > 1 were identified as differentially expressed based on the analysis.

### 2.7 Functional and Pathway Enrichment Analysis of Circular DNA‐Related Genes

Differentially expressed eccDNA‐related genes were subjected to functional enrichment analysis using Gene Set Enrichment Analysis (GSEA, http://www.gseamsigdb.org/gsea/msigdb/annotate.jsp). Kyoto Encyclopedia of Genes and Genomes (KEGG) and Gene Ontology (GO) pathway enrichment analyses were performed to elucidate biological functions and pathways. Visualization of eccDNAs was carried out using IGV software (Version 2.4.10) (Thorvaldsdottir et al. 2013).

### 2.8 Statistical Analysis

Statistical analyses were conducted using R‐4.1.2. For filtration of the over‐represented eccDNAs for crisp grass carp from the annotated eccDNAs, the significance of eccDNAs in the two groups was analyzed via R‐4.1.2 and assessed through Wilcoxon's rank‐sum test *p* value. Results were deemed statistically significant if the *p*‐value was less than 0.05.

## 3 Results

### 3.1 Detection of Linear DNA Removal and Circular DNA Enrichment

**FIGURE 1:**
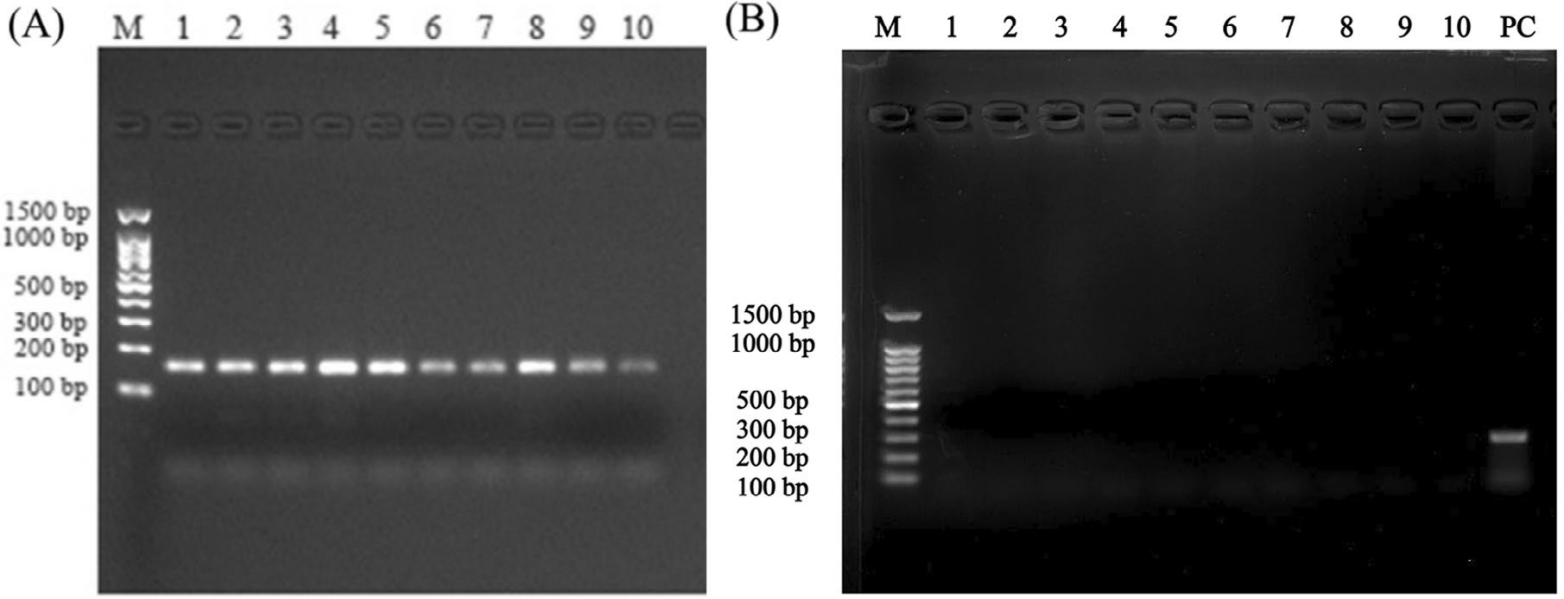
Electrophoresis results in the amplification of *COX3* (A) and *NDL6* (B) genes.

Electrophoresis results assessing the amplification of the *COX3* and *NDL6* genes, used to evaluate the removal of linear genomic DNA, are shown in Figure 1A,B, respectively. A distinct band was observed for *COX3*, while no band was detected for *NDL6*, indicating the complete elimination of linear genomic DNA.

**FIGURE 2:**
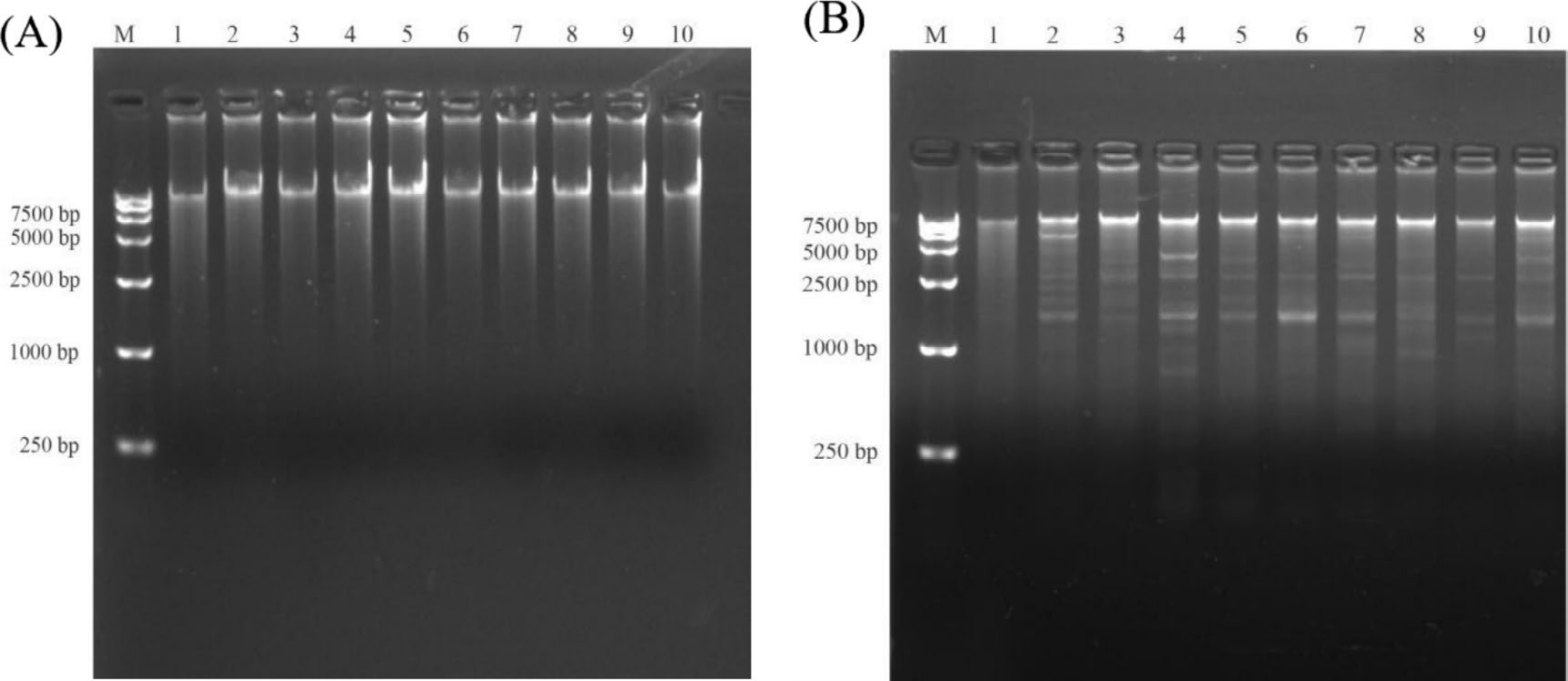
Electrophoresis results in the purification of eccDNAs after RCA reaction (A)and enzyme digestion verification (B).

Figure 2A,B depict electrophoresis results following the RCA reaction and subsequent enzyme digestion for eccDNA purification. The RCA reaction successfully concentrated intact eccDNAs, which, upon enzymatic digestion, yielded fragments of varying sizes.

### 3.2 Identification and Genomic Characteristics of eccDNA

Raw sequencing reads were generated using the Illumina NovaSeq 6000 platform. Data were processed using Trimmomatic to remove low‐quality reads (those containing ten or more unsequenced or low‐quality bases), PCR duplicates, and adapter sequences. The number of clean reads obtained from the six samples (crisp grass carp: 1–3; ordinary grass carp: 1–3) were as follows: 119,020,284 bp, 147,929,354 bp, 106,936,632 bp, 139,871,412 bp, 123,377,174 bp, and 130,942,792 bp, respectively (Table 1). EccDNA detection was performed using the Circle‐Seq approach on three samples from each of the crisp and ordinary grass carp groups. In total, 237,777 eccDNAs were identified, with 211,920 derived from crisp grass carp and 25,857 from ordinary grass carp. These results demonstrate that eccDNAs are ubiquitous in fish muscle and are significantly more abundant in crisp grass carp.

**TABLE 1:** Raw reads, clean reads and Q30 of samples.

Sample name	Raw reads	Clean reads	Q30 (%)
Crisp grass carp 1	135,401,198	119,020,284	90.75
Crisp grass carp 2	169,148,422	147,929,354	90.46
Crisp grass carp 3	121,513,794	106,936,632	89.95
Common grass carp 1	157,244,712	139,871,412	91.78
Common grass carp 2	139,295,000	123,377,174	91.32
Common grass carp 3	153,561,782	130,942,792	91.82

**FIGURE 3:**
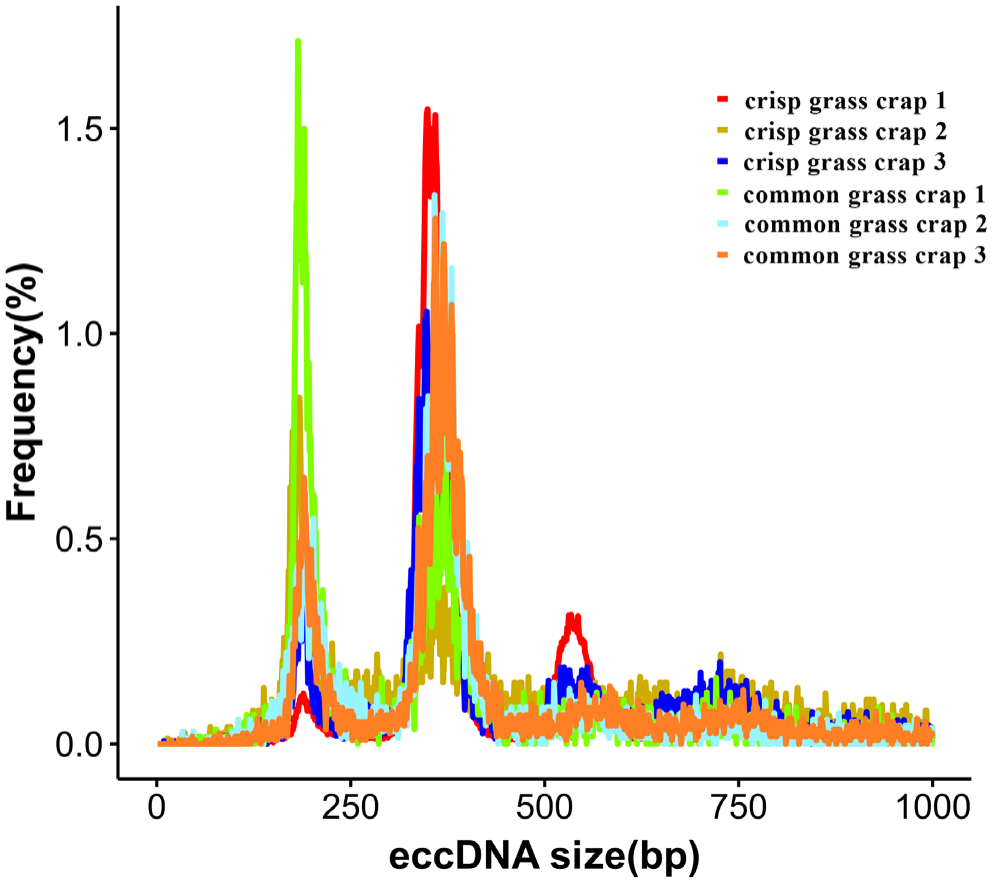
The length distribution of eccDNAs in two grass carp groups.

**FIGURE 4:**
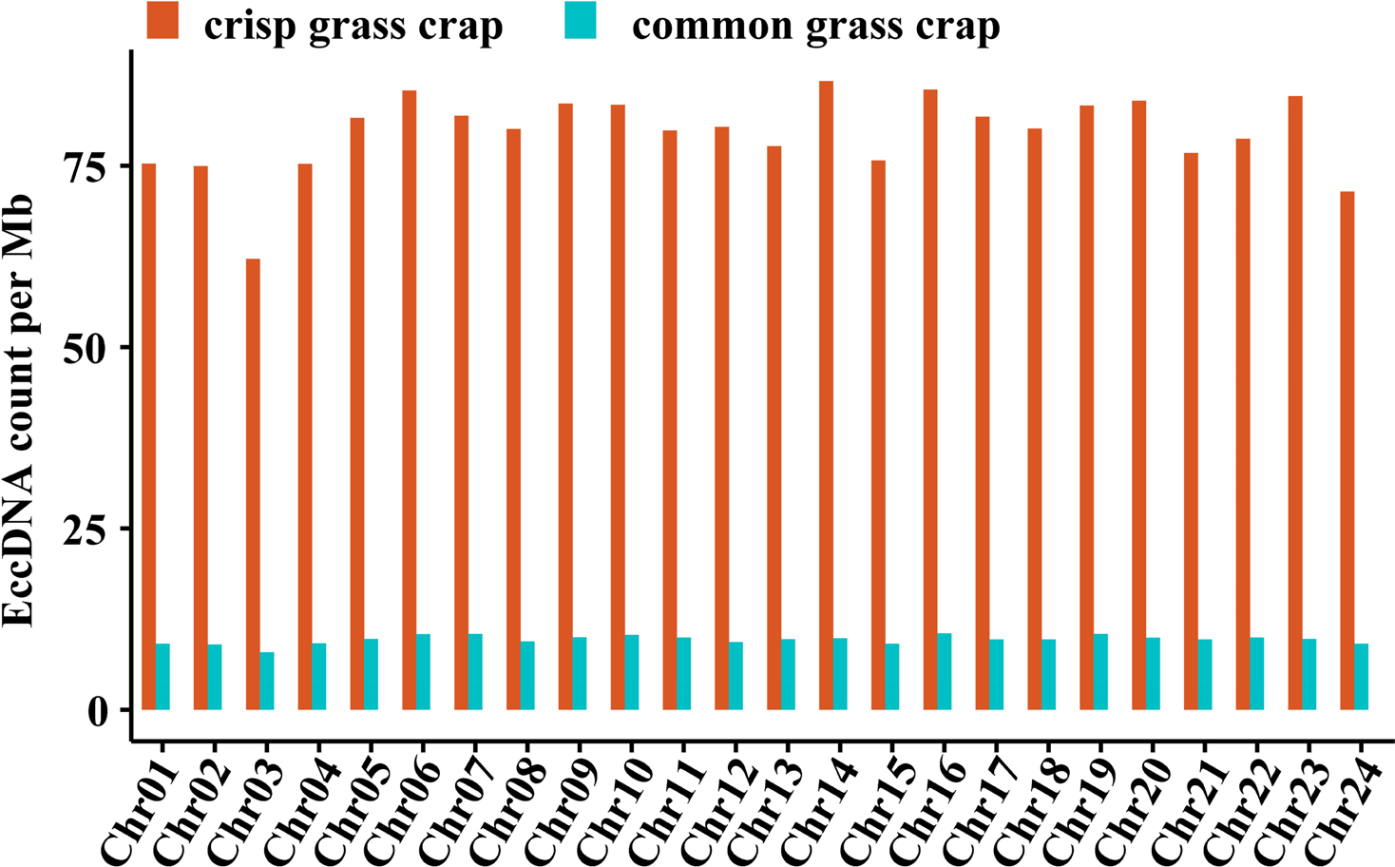
The distribution of eccDNAs in different chromosomes.

Analysis revealed that eccDNA lengths in grass carp muscle varied widely, predominantly ranging from 100 to 600 bp, with two peaks at approximately 185 bp and 300 bp (Figure 3). This size distribution aligns with findings from previous studies on muscle eccDNAs (Møller et al. 2020). EccDNA sequences in grass carp muscle were distributed across all chromosomes. Analysis of eccDNA frequency per megabase (Mb) on each chromosome showed that most chromosomes exhibited frequencies exceeding 75 eccDNAs per Mb. Notably, chromosomes 14, 16, and 23 displayed relatively higher frequencies compared to others (Figure 4).

**FIGURE 5:**
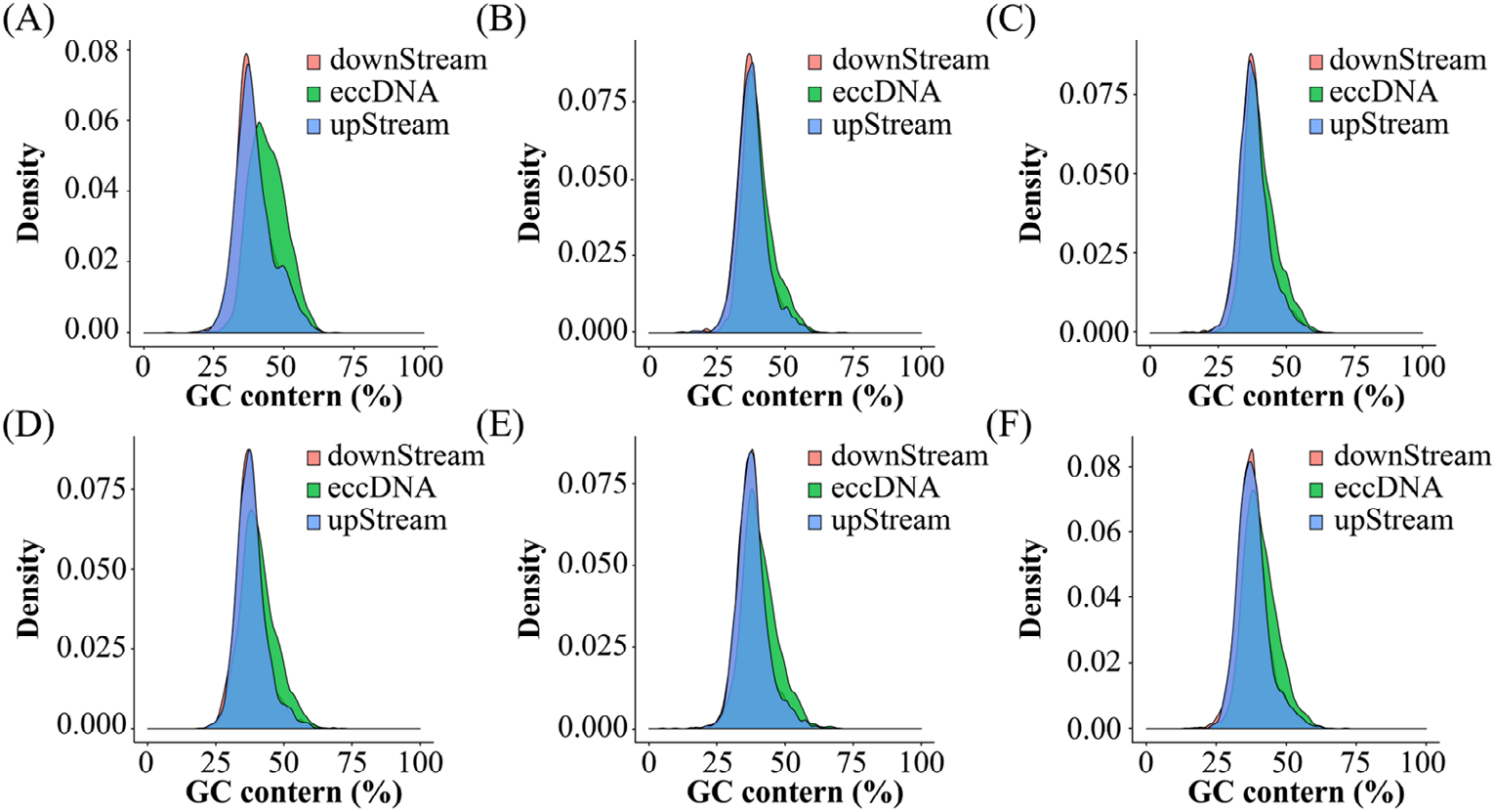
The GC content of eccDNA in crispy grass carp (A ~ C) and common grass carp (D ~ F).

Figure 5A–Fd illustrates the analysis of GC content within the eccDNA regions and their 1000 bp upstream and downstream flanking regions. The GC content in these regions ranged from 25% to 60%, with a peak at approximately 35%.

### 3.3 Differential Expression Profile and Annotation of eccDNAs

To investigate the molecular basis of fish fillet firmness, a comparative analysis of eccDNA expression profiles between the muscle tissues of crisp and ordinary grass carp was conducted. A total of 10,694 eccDNAs displayed differential expression between the two groups. Crisp grass carp exhibited 10,565 upregulated and 129 downregulated eccDNAs compared to ordinary grass carp. Annotation analysis revealed that upregulated eccDNAs were associated with 22,186 genes, whereas downregulated eccDNAs were linked to 59 genes.

### 3.4 GO and KEGG Enrichment Analysis of Differentially Expressed eccDNAs

**FIGURE 6:**
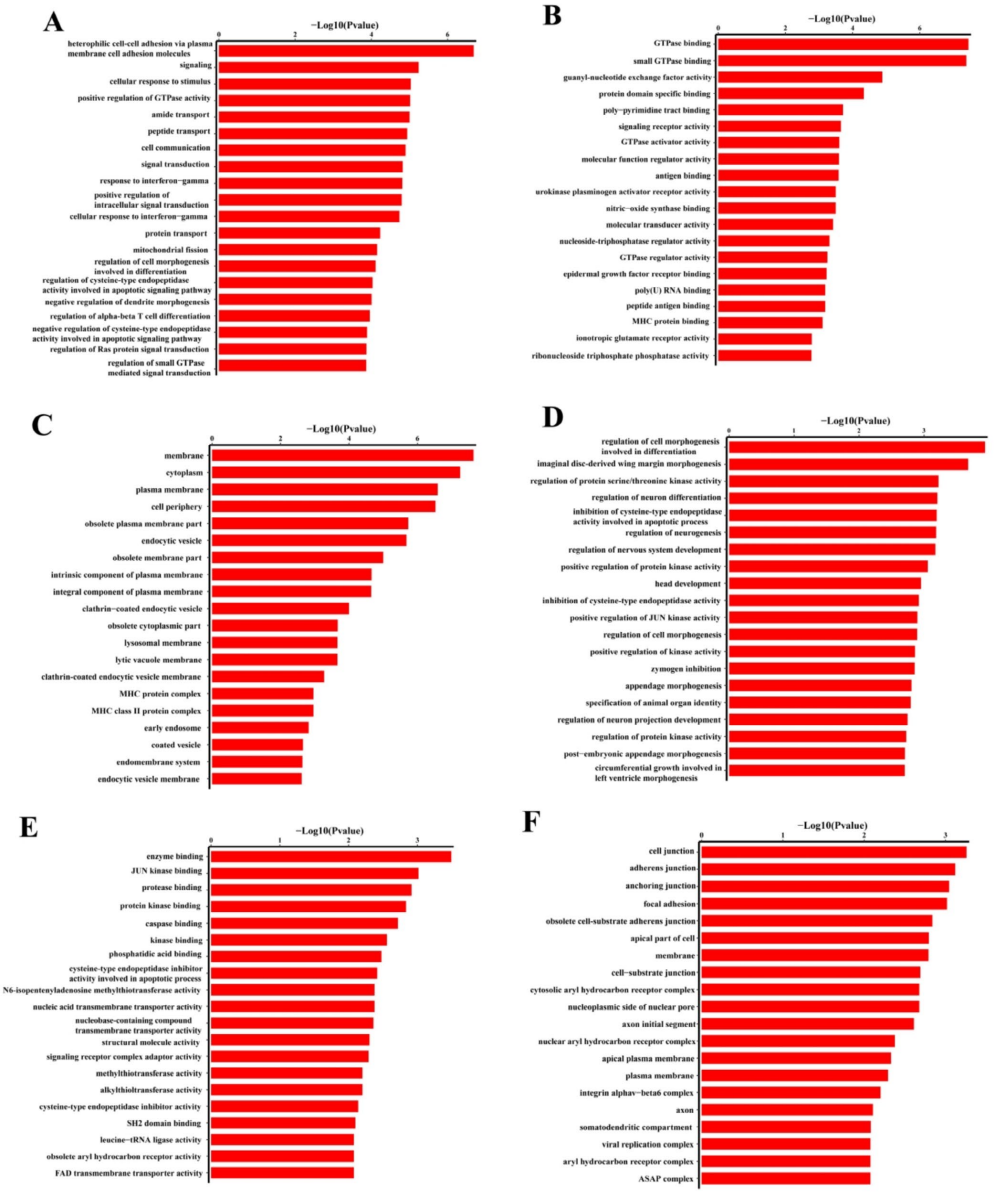
GO enrichment analysis of differentially expressed eccDNA‐associated genes. (A ~ C) referred to the GO enrichment analysis of up‐regulated eccDNAs in crisp grass carp, (A) Biological process, (B) Cellular component, (C) Molecular function; (D ~ F) referred to the GO enrichment analysis of down‐regulated eccDNAs in crisp grass carp, (D) Biological process, (E) Cellular component, (F) Molecular function.

GO enrichment analysis showed that genes corresponding to the upregulated eccDNAs were related to cellular components (guanyl‐nucleotide exchange factor activity, GTPase binding, and small GTPase binding and), biological processes (cellular and signaling response to stimulus) and molecular functions (membrane, cytoplasm and plasma membrane) (Figure 6A–C). For downregulated eccDNAs, associated genes were enriched in biological processes (regulation of cell morphogenesis during differentiation, imaginal disc‐derived wing margin morphogenesis and regulation of protein serine/threonine kinase activity), cellular components (JUN kinase binding, protease binding and enzyme binding) and molecular functions (cell junction, adherens junction and anchoring junction) (Figure 6D,E).

**FIGURE 7:**
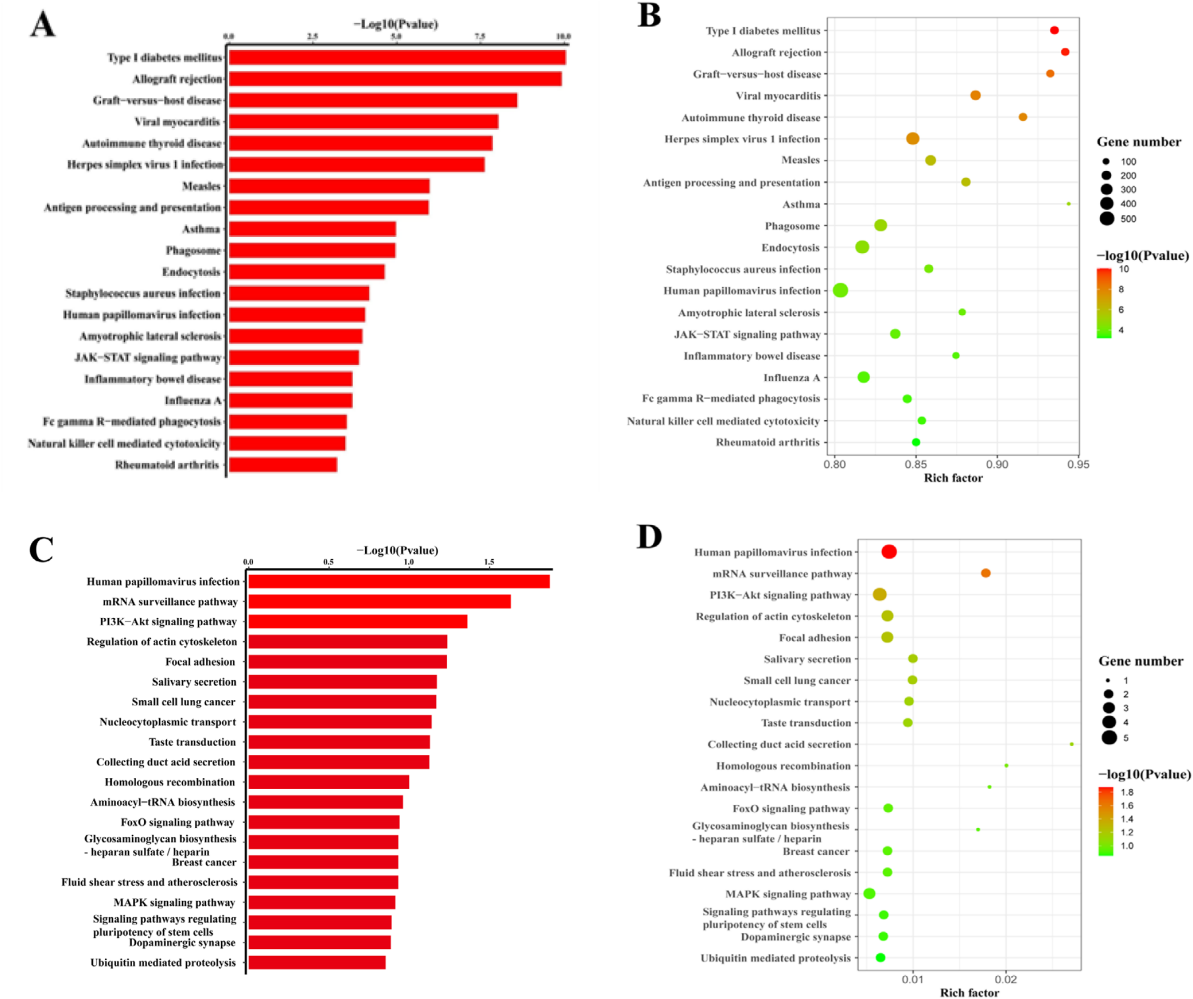
Pathway analysis of eccDNA‐associated genes. (A, B) referred to the up‐regulated pathways of eccDNAs; (C, D) referred to the down‐regulated pathways of eccDNAs.

Furthermore, KEGG pathway enrichment analysis identified 383 upregulated pathways associated with differentially expressed eccDNA‐related genes. These included pathways such as allograft rejection, type I diabetes mellitus, viral myocarditis, graft‐versus‐host disease, herpes simplex virus 1 infection, autoimmune thyroid disease, measles, antigen processing and presentation, asthma, and the JAK–STAT signaling pathway (Figure 7A–B). Conversely, 96 downregulated pathways were identified, including human papillomavirus infection, the mRNA surveillance pathway, and the PI3K‐Akt signaling pathway (Figure 7C,D).

### 3.5 Analysis of Differentially Expressed eccDNA‐Associated Genes Related to Muscle Hardness

Further analysis focused on differentially expressed eccDNA‐associated genes linked to muscle hardness in grass carp. Crisp grass carp displayed upregulated eccDNAs corresponding to genes implicated in muscle fiber structure, muscle development, cytoskeletal organization, and calcium metabolism. These included genes encoding collagen, talin, catenin, tubulin, and Myosin‐binding protein‐C (Table 2). These candidate eccDNAs likely contribute to the enhanced muscle hardness observed in crisp grass carp.

**TABLE 2:** Differentially expressed eccDNA‐associated genes involved in muscle fiber structure and development, cytoskeleton, collagen, and calcium metabolism of crisp grass carp.

Gene description	Annotation	Regulation	log_2_FC	*p*
Muscle fiber structure and development				
Myosin heavy chain	GC01Gene03301	Up	20.87947345	0.000000093
Myosin light chain	GC01Gene01667	Up	20.84801687	0.0000000972
Actin, alpha	GC01Gene01404	Up	21.40380748	0.0000000436
Actin‐related protein	GC01Gene01316	Up	10.0231253	0.010311326
Myosin binding protein	GC01Gene27142	Up	20.95861459	0.000000083
Myosin binding protein C, fast type a	GC01Gene04318	Up	10.32899188	0.008203815
Troponin C type 1b (slow)	GC01Gene27309	Up	8.33492794	0.032969156
Tropomyosin	GC01Gene03047	Up	20.84801687	0.0000000972
Nebulin repeat	GC01Gene02061	Up	21.47093962	0.0000000396
Cytoskeleton				
Tubulin	GC01Gene01232	Up	21.40380748	0.0000000436
Catenin	GC01Gene01138	Up	21.40380748	0.0000000436
Clathrin	GC01Gene14416	Up	8.522483704	0.029215294
Phospholipase C	GC01Gene02070	Up	21.47093962	0.0000000396
Capping protein	GC01Gene07223	Up	9.169432211	0.018953284
Nexilin (F Actin binding protein)	GC01Gene11576	Up	21.12714634	0.0000000652
Talin, middle domain	GC01Gene13969	Up	20.84801687	0.0000000972
Talin 2a	GC01Gene29803	Up	6.80371429	0.025924749
Collagen				
Collagen type I alpha	GC01Gene04089	Up	10.32899188	0.008203815
Collagen type II, alpha‐1a	GC01Gene11512	Up	21.12714634	0.0000000652
Calcium metabolism				
Calreticulin	GC01Gene03052	Up	20.84801687	0.0000000972
Calmodulin	GC01Gene16386	Up	8.745439622	0.025237536
Cadherin‐associated protein	GC01Gene01138	Up	21.40380748	0.0000000436
Annexin	GC01Gene00727	Up	21.12714634	0.0000000652
Protocadherin 10	GC01Gene00569	Up	21.12714634	0.0000000652
Rhomboid, veinlet‐like 3	GC01Gene08395	Up	8.059328516	0.039232168

## 4 Discussion

Muscle texture is a key determinant of fish product quality, directly influencing consumer preference and market value. A deeper understanding of the biological mechanisms underlying fish muscle texture can facilitate the development of high‐value products and enhance profitability in the aquaculture industry. Crisp grass carp, produced by feeding grass carp exclusively with faba beans, exhibit superior muscle texture compared to ordinary grass carp. This enhanced textural quality includes increased chewiness, firmness, gumminess, shear force, and springiness, making crisp grass carp a premium aquaculture product (Chen et al. 2020). Previous research has primarily focused on understanding the mechanisms that enhance muscle texture in grass carp, with the goal of producing high‐quality fish fillets. This study represents a significant advancement, as it is the first to compare eccDNA profiles between the muscle tissues of crisp and ordinary grass carp, providing valuable insights for future research on fish fillet firmness.

ROS like hydrogen peroxide (H_2_O_2_) is produced by the two glucosidic aminopyrimidine derivatives (vicine and convicine) that are in abundance in faba beans (Winterbourn et al. 1986). Metabolomics studies report significantly elevated ROS levels (up to four times) in the skeletal muscle of crisp grass carp (Yu et al. 2020). ROS are known to play key roles in regulating muscle metabolism and are likely to influence muscle texture (Archile‐Contreras and Purslow 2011). However, while faba beans improve muscle texture, they also hinder growth and may induce nutritional deficiencies and oxidative damage (Ma et al. 2020; Fu et al. 2022). In this study, we observed that the abundance of eccDNAs in crisp grass carp muscle (211,920) was significantly higher than in ordinary grass carp muscle (25,857). Moreover, 10,823 differentially expressed eccDNAs were identified between crisp grass carp muscle and ordinary grass carp muscle, including 10,694 upregulated and 129 downregulated eccDNAs in the muscle of crisp grass carp. Increased oxidative stress has been shown to exacerbate chromosomal damage, leading to both double‐strand breaks (DSB) and single‐strand breaks (SSB), as well as promoting base oxidation (Dutta et al. 2015; Cannan and Pederson 2016). These processes facilitate the formation of circular DNA molecules, which may explain the higher abundance of eccDNAs observed in crisp grass carp. We propose that the elevated oxidative stress induced by faba bean feeding facilitates the detachment of DNA from chromosomes and its subsequent circularization in fish muscle.

The quality of fish meat and muscle mass is profoundly influenced by two critical factors: the size and number of muscle fibers (Tang et al. 2024). Previous findings have demonstrated that muscle fibers are closely related to increased fish muscle hardness, and faba bean‐induced muscle growth in grass carp results from hyperplasia, characterized by a lifelong increase in myofiber number (Johnston et al. 2000). Myofibrils, the functional units of muscle fibers, primarily consist of thin filaments (comprising troponin proteins, actin, and tropomyosin), thick filaments (containing myosin proteins), titin, the M‐line, and the Z‐disk (composed of nebulin proteins and desmin) (Nishimura 2010). Myofibrillar proteins constitute the predominant protein class in skeletal muscle, accounting for around 60% of the total protein composition by weight (Huang et al. 2012). Previous findings showed that genes associated with myofibroblast proliferation and cytokine production were upregulated at both the mRNA level and protein level in crisp grass carp compared to ordinary grass carp (Yu, Xie et al. 2014; Yu, Liu et al. 2014; Yu et al. 2017; Xu et al. 2020). Coincidentally, in the present study, functional enrichment analysis of the upregulated eccDNAs in crisp grass carp revealed significant associations with genes related to muscle fiber structure, muscle cell development, and differentiation. These included genes encoding capping protein, myosin, tropomyosin, talin, annexin, catenin, clathrin, tubulin, and others. These findings suggest that the molecular regulation of muscle hardening is substantially influenced by upregulated eccDNAs enriched in muscle‐related functions. The enhanced chromosomal and extrachromosomal gene activity, high copy number, and increased transcriptional activity of eccDNAs likely contribute to gene overexpression (Paulsen et al. 2018; Zhu et al. 2021). Therefore, it is plausible that the overexpression of muscle fiber‐associated genes driven by eccDNAs contributes to increased muscle fiber density, thereby enhancing muscle hardness.

Additionally, crisp grass carp muscle contains higher collagen (Ma et al. 2020; Tang et al. 2024). Notably, compared to ordinary grass carp muscle, the eccDNAs of type I and type II collagen genes were significantly more abundant in crisp grass carp muscle, aligning with this observation. Interestingly, previous studies have shown that collagen genes were highly expressed at both the mRNA level and protein level in crisp grass carp muscle (Yu, Xie et al. 2014; Yu, Liu et al. 2014; Yu et al. 2017). In grass carp‐fed faba beans, genes encoding type I and type II collagen appear to play a pivotal role in muscle hardening (Yu, Xie et al. 2014; Yu, Liu et al. 2014). These findings indicate that the increased skeletal muscle firmness in crisp grass carp is likely attributable to the elevated eccDNA levels of collagen genes.

The density of filamentous myosin is known to be upregulated by calcium (Herrera et al. 2002). Moreover, increased filamentous myosin density has been linked to increased muscle firmness (Hatae et al. 1990). Notably, previous studies reported higher filamentous myosin density and elevated calcium content (Liu et al. 2011) in crisp grass carp compared to ordinary grass carp, which helps elucidate the mechanism underlying the increased muscle firmness observed in crisp grass carp (Lin et al. 2009). Additionally, numerous genes associated with calcium metabolism, such as cadherin protein (Cad), calreticulin (CRT) and calmodulin (CaM), were upregulated at both the mRNA level and protein level in crisp grass carp (Yu, Xie et al. 2014; Yu, Liu et al. 2014; Yu et al. 2017). Additionally, the eccDNAs of CRT and CaM were abundant in the muscle cell of slimming grass carp (He et al. 2024). Consistently, eccDNAs of CRT, CaM, and Cad were also upregulated in crisp grass carp, indicating that the eccDNA of calcium‐dependent protein genes partially contribute to the elevated calcium levels, which contribute to increased muscle firmness.

Recent studies revealed that eccDNA is a potent innate immunostimulant, with a strong ability to induce cytokine production (Wang et al. 2021; Zuo et al. 2022). Purified eccDNAs or synthetic circular DNA exhibit much stronger immunostimulatory effects compared to their linear counterparts, significantly enhancing the expression of immune‐related genes (Wang et al. 2021). In line with this, our analysis revealed that the majority of upregulated eccDNA‐related genes in crisp grass carp were enriched in immune‐related classes, including allograft rejection, type I diabetes mellitus, graft‐versus‐host disease, JAK–STAT signaling pathway, and viral myocarditis. These pathways are closely related to immune‐mediated diseases, inflammation, immune response, oxidative stress, cell apoptosis, and autophagy (Seif et al. 2017; Abboud et al. 2020; Eizirik et al. 2020; Lasrado and Reddy 2020), indicating that eccDNA may play a role in triggering immune responses in fish. If future studies confirm this hypothesis, the eccDNAs identified in this study could provide a foundation for developing fish vaccines and immunotherapeutic strategies.

## 5 Conclusions

In conclusion, using the Circle‐seq strategy and rigorous pipeline analysis, we generated a new and basic eccDNA database for the molecular regulation of fish muscle firmness. During the increase in muscle firmness, a total of 10,757 eccDNAs were found to be differentially expressed in crisp grass carp compared to ordinary grass carp. These eccDNAs were mainly enriched in functional groups that played key roles in the molecular regulation of muscle hardening. EccDNA may also function as a regulatory elements that regulate gene amplification and expression. Future studies should focus on illuminating the molecular mechanisms by which eccDNAs influence muscle firmness and investigating their potential to improve muscle texture in cultured fish.

## Author Contributions

**Kai Zhang:** formal analysis (lead), funding acquisition (lead), investigation (lead), methodology (lead), visualization (lead), writing – original draft (lead), writing – review and editing (lead). **Jianchao Chen:** data curation (equal), investigation (equal), methodology (equal). **Haobin He:** data curation (equal), investigation (equal). **Binwei Duan:** data curation (equal), investigation (equal), methodology (equal). **Canbei You:** data curation (equal), investigation (equal), methodology (equal). **Zehua Hu:** data curation (equal), investigation (equal). **Linhao Cai:** data curation (equal), investigation (equal). **Xi Xiang:** supervision (lead), writing – review and editing (lead). **Rishen Liang:** funding acquisition (lead), supervision (lead), writing – review and editing (lead).

## Conflicts of Interest

The authors declare no conflicts of interest.

## Data Availability

The study data are available in the article.
